# Atopic dermatitis-associated genetic variants regulate LOC100294145 expression implicating interleukin-27 production and type 1 interferon signaling

**DOI:** 10.1016/j.waojou.2023.100869

**Published:** 2024-01-12

**Authors:** Wei Yi Teo, Yi Ying Eliza Lim, Yang Yie Sio, Yee-How Say, Kavita Reginald, Fook Tim Chew

**Affiliations:** a Department of Biological Sciences, National University of Singapore, Singapore; b Department of Biomedical Science, Faculty of Science, Universiti Tunku Abdul Rahman (UTAR) Kampar Campus, Kampar, Perak, Malaysia; c Department of Biological Sciences, School of Medical and Life Sciences, Sunway University, Malaysia

**Keywords:** Atopic dermatitis, Genome-wide association study, Long non-coding RNA, Mendelian randomization, Multi-omics

## Abstract

**Background:**

Atopic dermatitis (AD) is a complex inflammatory disease with a strong genetic component. A singular approach of genome wide association studies (GWAS) can identify AD-associated genetic variants, but is unable to explain their functional relevance in AD. This study aims to characterize AD-associated genetic variants and elucidate the mechanisms leading to AD through a multi-omics approach.

**Methods:**

GWAS identified an association between genetic variants at 6p21.32 locus and AD. Genotypes of 6p21.32 locus variants were evaluated against *LOC100294145* expression in peripheral blood mononuclear cells (PBMCs). Their influence on *LOC100294145* promoter activity was measured *in vitro* via a dual-luciferase assay. The function of *LOC100294145* was then elucidated through a combination of co-expression analyses and gene enrichment with g:Profiler. Mendelian randomization was further used to assess the causal regulatory effect of *LOC100294145* on its co-expressed genes.

**Results:**

Minor alleles of rs116160149 and rs115388857 at 6p21.32 locus were associated with increased AD risk (*p* = 2.175 × 10^−8^, OR = 1.552; *p* = 2.805 × 10^−9^, OR = 1.55) and higher *LOC100294145* expression in PBMCs (*adjusted p* = 0.182; 8.267 × 10^−12^). *LOC100294145* expression was also found to be increased in those with AD (*adjusted p* = 3.653 × 10^−2^). The genotype effect of 6p21.32 locus on *LOC100294145* promoter activity was further validated *in vitro*. Co-expression analyses predicted LOC100294145 protein's involvement in interleukin-27 and type 1 interferon signaling, which was further substantiated through mendelian randomization.

**Conclusion:**

Genetic variants at 6p21.32 locus increase AD susceptibility through raising *LOC100294145* expression. A multi-omics approach enabled the deduction of its pathogenesis model comprising dysregulation of hub genes involved in type 1 interferon and interleukin 27 signaling.

## Introduction

Atopic dermatitis (AD) is a chronic inflammatory skin disease characterized by itch, dryness, and eczematous lesions. In developed countries, the prevalence of AD has increased, estimated to be 1–3% and 15–20% in adults and children respectively.^1^ Patients with moderate to severe AD are more likely to have comorbidities like such as fever, asthma, and food allergies. Chronic loss of sleep and persistent itch in AD patients also lead to a poorer quality of life alongside an increased risk of mental disorders.^2^ It is therefore critical to uncover the etiology of AD for the improvement of treatment strategies.

The heritability of AD was estimated to be 75% from twin studies, where the concordance ratio of monozygotic twins is 3 times higher than dizygotic twins.^3^ It was found that the strongest risk factor for AD is a family history of atopic disease and that a person's risk of AD increases by 3 to 5 times when 1 or both of their parents had AD.^4^^,^^5^ As such, numerous genetic studies have been conducted to investigate the molecular mechanisms leading to AD pathology. To date, genome-wide association studies (GWAS) have successfully identified more than 30 genetic loci that influence AD.^6^ Disease susceptibility genes found in these loci are commonly involved in immunological and skin barrier defects.^7^, ^8^, ^9^

Despite the success of GWAS in identifying disease-associated genes, less than 20% of AD's heritability has been explained by GWAS thus far;^6^^,^^10^ the discrepancy between the expected heritability and the proportion of genetic risk currently explained by literature is known as the "missing heritability problem". The singular approach of GWAS is insufficient to fully explain the genetic etiology and molecular pathways of complex traits, prompting the integration of multi-omics data to account for the missing heritability.^6^^,^^11^^,^^12^ A widely used multi-omics strategy called the triangle method integrates data from GWAS, expression quantitative trait loci (eQTL) and differential gene expression analyses.^13^ Here, disease-associated single nucleotide polymorphisms (SNPs) are first filtered by GWAS. These SNPs are then tested for association with gene expression through an eQTL analysis. Lastly, the expression of shortlisted genes from the previous step are tested for association with the disease. The power of this method lies in the rationale that a genetic mutation first perturbs gene expression level, which then influences the disease phenotype. As such, this approach aims to identify functional SNPs which influence the complex trait by altering the expression levels of disease susceptibility genes. Previous integration of gene expression data allowing fine-mapping to the causal genetic variants of interest has enhanced existing knowledge in the pathogenesis of various diseases.^14^^,^^15^ The incorporation of genetic and functional data in multi-omics analyses therefore allows for an improved understanding of underlying biological pathways in AD's complex pathogenesis.

**Fig. 1:**
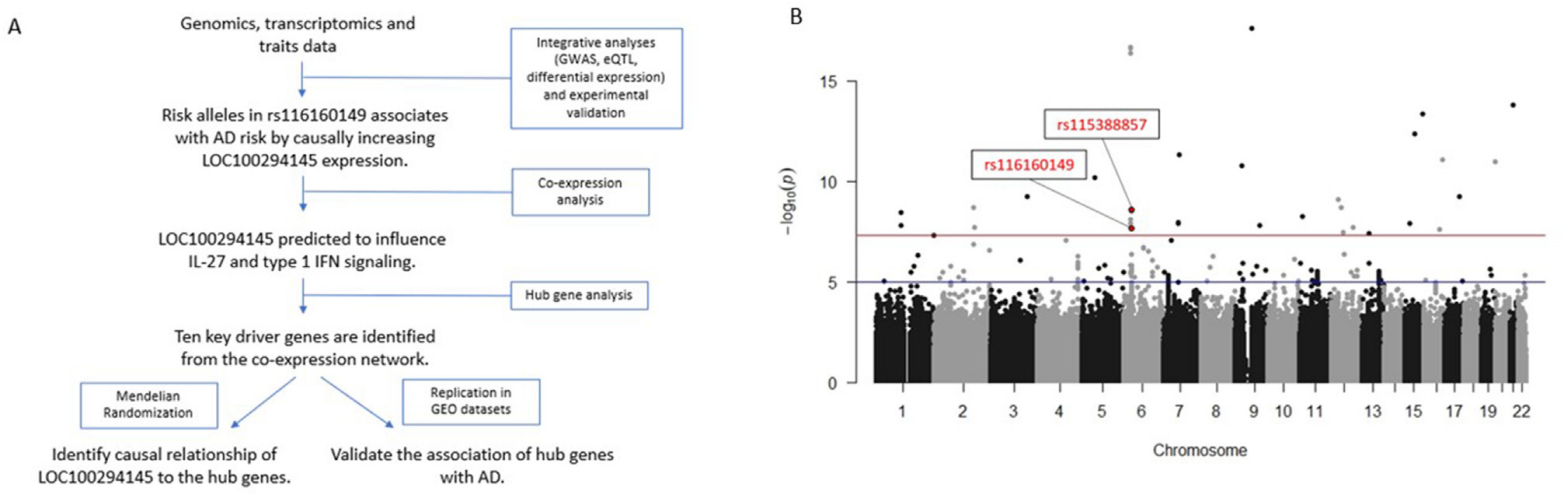
(A) Workflow of the study. *LOC100294145* is first identified as the gene of interest with an integrative analysis. Its functions are then elucidated via various downstream analyses. (B) Manhattan plot showing the associations between all SNPs and the AD phenotype (AD cases vs non-atopic non-AD controls) after adjustment for age, gender and the first 10 principal components. Y-axis shows the p-value in log scale and x-axis shows the chromosomal position of each SNP. The red and the blue line represent the GWAS significant (*p* < 5 × 10^−8^) and GWAS suggestive threshold (*p* < 1 × 10^−5^) respectively

Here we integrated GWAS, expression quantitative trait loci (eQTL) and differential gene expression to identify single nucleotide polymorphisms (SNPs) and genes of interest. *LOC100294145*, containing SNP rs116160149 (*p* = 2.175 × 10^−8^, OR = 1.552) and SNP rs115388857 (*p* = 2.805 × 10^−9^; OR = 1.55) in its promoter at 6p21.32 locus, was selected for downstream studies following this integrated systems-level study of AD. Increased *LOC100294145* expression in PBMCs was found to be associated with the risk alleles of SNPs rs116160149, rs115388857, and with AD cases. We also showed that the risk alleles increased *LOC100294145* promoter activity *in vitro*. We predicted that *LOC100294145* is functionally involved in interleukin-27 (IL-27) and type 1 interferon (IFN) signaling and strengthened this hypothesis through a mendelian randomization study. Due to the potential of IL-27 and type 1 IFN signaling in eliciting autoallergy, serum IgE levels in our study's cohort was also assessed. The overall workflow of this study is illustrated in Fig. 1A.

## Materials and methods

### Genome wide association studies

The GWAS cohort consisted of 1679 Singapore Chinese recruited from the National University of Singapore (NUS). Plink 1.9 was used to analyze genotype data extracted from buccal cells. The genotype-phenotype associations were measured with p-values of logistic regression and odds ratios with 95% CI, adjusted for age, gender and the first 10 principal components. AD cases and controls were classified accordingly by trained personnel as per validated guidelines from the UK Working Party Criteria and Hanifin & Rajka Criteria.^16^^,^^17^ The clinical symptoms of AD were defined to be a recurrent flexural itch lasting 6 months or longer. A house dust mite skin prick test was performed alongside to define atopy status, which showed high sensitivity and specificity in previous studies when compared to the clinical standard employing presence of allergic co-morbidities.^18^ AD cases were thus defined as subjects with AD symptoms and a positive atopic status which is described in detail in Lim et al. Atopic and non-atopic controls have a positive and negative atopic status respectively with no AD symptoms. Functional prediction of identified SNPs was performed using regulomeDB and SNPinfo database.^19^^,^^20^ Linkage disequilibrium (LD) was determined using plink 1.9, where an R^2^ value of 0.8 is defined as a significant LD.^21^ The genotyping procedure and definition of atopic status are documented in the supplementary text.

### Transcriptome regulation in peripheral blood mononuclear cells (PBMCs)

The RNA expression in peripheral blood mononuclear cells (PBMCs) was measured in 300 individuals from Singapore and Malaysia. PBMCs were first isolated from whole blood collected from each subject, and total RNA was extracted for whole-transcriptome sequencing as documented in the supplementary text. Genes within a 10 kb flanking region of disease-associated SNPs are included in the expression quantitative trait loci analysis to investigate the association between genotype and RNA expression. The association of RNA expression in PBMCs with the disease or genotype was analyzed using ANOVA and independent sample *t*-test.

### Promoter luciferase assay

Luciferase assay was used to evaluate the haplotype effect of *LOC100294145* promoter SNPs in HEK293 cells. The region −1 to −2000 bp of the *LOC100294145* gene was cloned into the promoter-less luciferase plasmid pGL4.10 (Promega). Human embryonic kidney cells (HEK293) obtained from the American Type Culture Collection (ATCC) were cultured in Dulbecco's modified Eagle medium (DMEM) (Sigma-Aldrich) containing 1% l-glutamine and 10% fetal bovine serum (Thermo Scientific). HEK293 cells were cultured in an incubator maintained at 37 °C and 5% CO2 and transfected at 80% confluency. Lipofectamine 2000 (Invitrogen) was used to transiently transfect HEK293 cells with the plasmid constructs. Normalization to control for transfection efficiency variation was performed through co-transfection with renilla luciferase control plasmid pGL4.74. Luciferase activity was determined using the Dual-Luciferase® Reporter Assay System kit (Promega) and Promega™ GloMax® Luminometer (Promega) according to manufacturer's instructions. Promoter activity was calculated by dividing the Firefly luciferase activity by the Renilla luciferase activity and presented as Relative Luciferase Units (RLU). The transfection experiments were performed in triplicates with HEK293 cells harvested at 24 h and 48 h post-transfection. Independent sample *t*-test was used to evaluate the association between luciferase promoter activity level with the various haplotypes.

### Differential Co-expression analysis

Correlation of RNA expression levels was calculated between *LOC100294145* and 12,775 known expressed coding genes in a co-expression analysis. The Benjamini-Hochberg tool was applied to obtain false discovery rate (FDR) adjusted p-values. Using Pearson's product moment correlation coefficient, an FDR-adjusted p-value of less than 0.05 was used to identify significantly co-expressed genes. Differentially co-expressed genes were then selected by comparing gene expression between AD cases, atopic and non-atopic controls (FDR-adjusted *p* < 0.05, |log-2 fold change| > 1).

### Protein-protein interaction co-expression network construction

A protein-protein interaction (PPI) network of differentially co-expressed genes was constructed using the STRING database.^22^ The Molecular Complex Detection (MCODE) plugin in Cytoscape was then used to cluster the protein interactions in the network using a node score cutoff of 0.2, a module score of >4, a degree cutoff of 2 and a maximum depth of 100.^23^, ^24^, ^25^, ^26^, ^27^ Next, the Cytohubba plug-in in Cytoscape was used to identify biologically important hub genes that are highly connected via protein-protein interactions as documented in the supplementary text.^28^^,^^29^

### Gene enrichment analysis

The enriched biological functions of the selected PPI subnetwork and hub genes were analyzed with g:Profiler, based on the Gene Ontology: Biological Processes (GO:BP) and Kyoto Encyclopedia of Genes and Genomes (KEGG) databases.^30^ The receiver operating characteristic area under the curve (ROC AUC) was then used to evaluate the diagnostic value of the hub genes against AD.

### Validation using gene expression omnibus (GEO) datasets

We downloaded 4 RNA expression datasets (GSE5667, GSE32924, GSE27887, GSE32473) from the gene expression omnibus datasets (GEO) database to replicate the dysregulation of the identified hub genes in other studies.^31^, ^32^, ^33^, ^34^, ^35^, ^36^ The GEO2R tool was used to obtain significant DEGs (*p* < 0.05) from the various datasets.^31^

### Mendelian randomization

Mendelian randomization (MR) was employed to evaluate the causal relationship of *LOC100294145* to the expression of hub genes. SNPs that are associated with the expression of *LOC100294145* were chosen as instrumental variables (*p* < 1 × 10^−5^), after clumping to remove SNPs that are in linkage disequilibrium (LD > 0.1). We identified 45 independent SNPs to be used as genetic instruments. The F-statistic of the instrumental variables used is more than 10 to prevent weak instrument bias. The first 10 principal components were used to control for population stratification.

MR was performed using 4 different methods: inverse-variance weighted (IVW), simple median, weighted median and the MR-Egger method. The IVW method was performed as the primary method, which is a weighted sum of the causal estimates obtained from each instrumental SNP. The simple median method selects the median causal estimate out of what was estimated by all instrumental SNPs, obtaining consistent results if up to 50% of the SNPs are valid and strong instruments. The aforementioned methods do not account for pleiotropy; thus, weighted median and MR-Egger which can account for pleiotropy are performed as well. The weighted median method can tolerate pleiotropy as it gives a consistent estimate if up to 50% of the weight comes from valid instruments. MR-Egger allows pleiotropy to be measured with its intercept term. Lastly, MR-PRESSO (Mendelian Randomization Pleiotropy RESidual Sum and Outlier) is used to identify influential SNPs which are removed in a stepwise manner and re-evaluated using the 4 methods mentioned. In summary, MR analyses is performed in 3 steps: the first step involves MR analyses with all instrumental SNPs, while the second and third step involve removal of instrumental SNPs with a p-value of less than 0.05 and 1 from the MR-PRESSO outlier test respectively. The analyses are performed using the R packages “Mendelian Randomization” and “MRPRESSO”.

### Autoallergy evaluation by serum IgE levels

Serum was separated from whole blood obtained from 111 participants in our cross-sectional cohort. IgE response to 7 human fungal homolog allergens (Human Enolase ID: NM_001428.5; Human thioredoxin ID: X77584; Human cyclophilin ID: NM_000942; Human heat shock protein ID: NM_006597.6; Human alcohol dehydrogenase ID: AAV38636.1; Human aldehyde dehydrogenase ID: AAA51693.1; Human 60S acidic ribosomal protein P2 ID: AJ224333) was screened using an immuno-dot blot assay as described by Kidon et al. (2011).^37^ IgE titers greater than 3.5 kU/L (Class 3 and above) were considered as positive autoallergy. All immuno-dot blot measurements were batch normalized.

## Results

### Integrative analysis of GWAS, eQTL and differential expression identified long non-coding RNA *LOC100294145* as a gene of interest

The genetic association cohort consisted of 1679 Singapore Chinese (956 AD cases and 723 non-atopic controls), with a mean age of 21.7 ± 4.9 (Table S1). In accordance with the triangle method discussed previously, we first performed GWAS and identified 35 trait-associated SNPs (GWAS *p* < 5x10^−8^) after adjustment for age, gender, and the first 10 principal components (Fig. 1B; Table S2). The genomic inflation factor was 1.086, suggesting little evidence of population stratification (Figure S2).

Studies have shown that the statistically associated SNPs are often in linkage disequilibrium (LD) with other true disease-causing variants.^38^^,^^39^ Therefore, to identify regulatory genetic variants that can perturb disease susceptibility genes, we included an additional biological constraint where only AD-associated SNPs (or their LD SNPs) that are functionally annotated with alterations to transcription factor binding sites (TFBS), splice sites or 3′-untranslated regions are included for further analyses. Through *in silico* predictions using SNPinfo and regulomeDB,^19^^,^^20^ 7 AD-associated SNPs near 6 genes (consisting of 12 unique transcripts) were annotated with regulatory functions (Table S3).

Since disease-associated variants found to be enriched in eQTLs are likely to be regulatory SNPs,^40^ we sought to identify AD-associated genetic variants that are also functionally associated with gene expression out of these shortlisted SNPs. As such, we performed an eQTL analysis using PBMC samples from the functional association cohort (n = 278) and obtained 8 significant eQTL associations (adjusted *p* < 0.05) consisting of 5 SNPs and 4 genes (Table S3). Finally, expression levels of these genes were compared across AD phenotypes to pinpoint differentially expressed genes, identifying *LOC100294145* as a gene of interest (adjusted *p* = 0.0365; Table S3).

### Promoter SNPs rs116160149 and rs115388857 in *LOC100294145* overlap transcription factor binding sites and increases the risk of AD

SNPs rs116160149 and rs115388857 are located at 6p21.32 locus, approximately 300 bp upstream of the *LOC100294145* gene. In the genetic association cohort (n = 1679), an increased risk of AD was found to be associated with the minor allele “T” of rs116160149 (*p* = 2.175 × 10^−8^; OR = 1.552; 95% CI = 1.33–1.81) and the minor allele “G" of rs115388857 (*p* = 2.805 × 10^−9^; OR = 1.55; 95% CI = 1.341–1.791) (Table S2). Predictions *in silico* also functionally annotated rs116160149 and rs115388857 as regulatory SNPs that overlap a TFBS in the promoter region of the *LOC100294145* gene. Based on reported whole blood ChIP-seq data,^19^ transcription factors *BHLHE40*, *USF1*, and *RUNX3* were predicted to bind to the promoter region of *LOC100294145* (Figure S3A, Table S4). Frequency matrix profile of transcription factors *BHLHE40* and *USF1* were also in concordance with the hypothesis (available at JASPER, https://jaspar.genereg.net/). Affinity for the “T” allele of rs116160149 and “G” allele of rs115388857 were associated with higher *LOC100294145* transcript expression levels (Figure S3B). The transcription factors of *LOC100294145* are well-characterized in their immune functions, such as in antigen presentation,^41^, ^42^, ^43^, ^44^ differentiation of memory cytotoxic T cells,^44^ and gene regulation in various immune cell types.^45^^,^^46^ As transcription factors modulate genes of related functions, *LOC100294145* is likely to modulate similar immune-related processes. Due to the putative involvement of immunity in AD, this study focuses on characterizing *LOC100294145* polymorphisms and its biological functions.

### rs116160149 and rs115388857 are eQTLs for *LOC100294145* and *LOC100294145* is differentially expressed between AD phenotypes in PBMCs

**Fig. 2:**
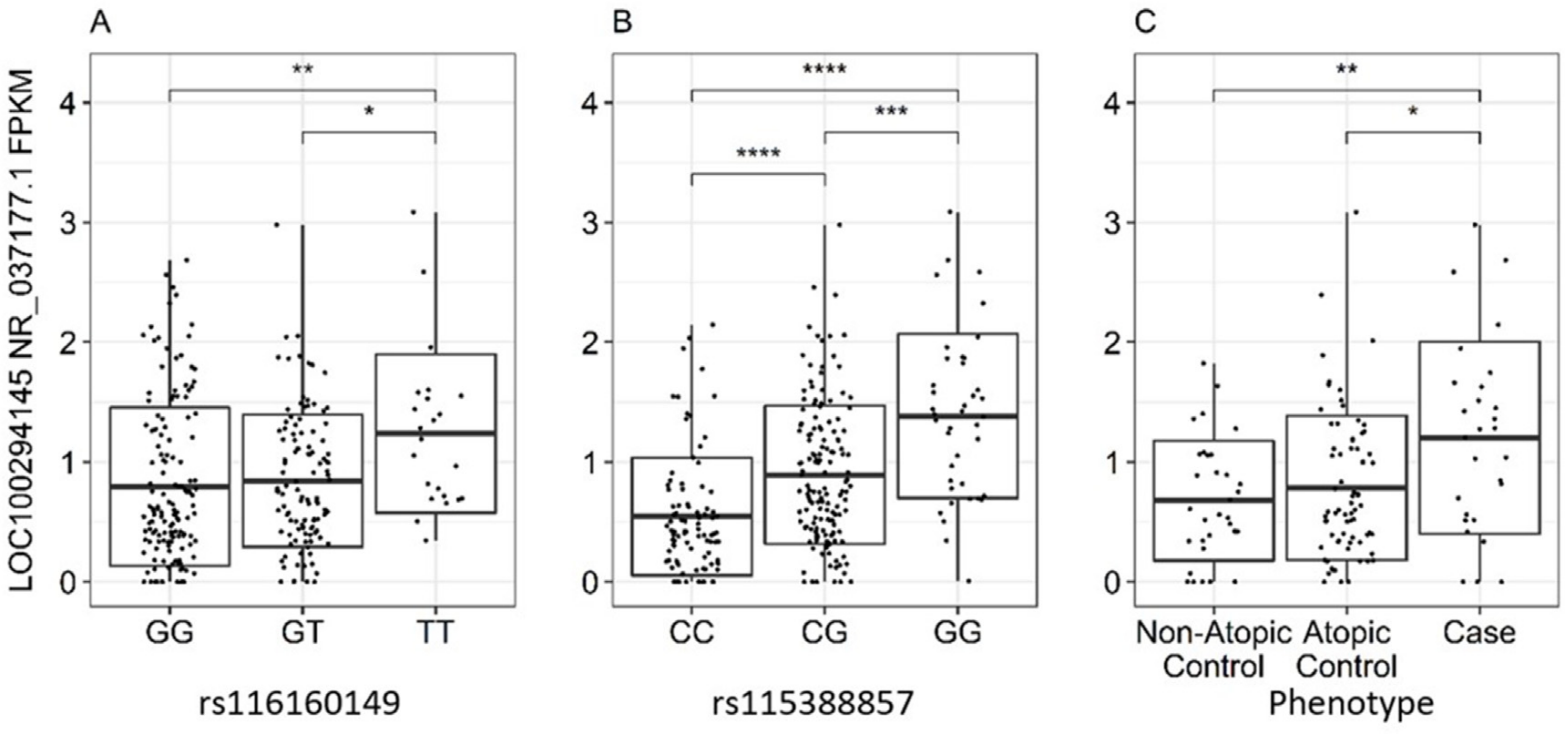
(A) eQTL showing the association between rs116160149 genotypes and *LOC100294145* expression in the functional association cohort. The risk genotype of rs116160149 is TT. (B) eQTL showing the association between rs115388857 genotypes and *LOC100294145* expression in the functional association cohort. The risk genotype of rs115399957 is GG. (C) Differential gene expression analyses showing the association between AD phenotypes and *LOC100294145* expression in the functional association cohort (∗*p* < 0.05, ∗∗*p* < 0.01, ∗∗∗*p* < 0.001, ∗∗∗∗*p* < 0.0001)

In the integrative analysis, we further performed eQTL and differential gene expression analysis following the triangle method. Transcript expression data of 278 PBMC samples from the functional association cohort (Table S1) were used to identify associations between the risk variants and *LOC100294145* expression. The minor genotype “TT”, of rs116160149 showed an eQTL dosage effect with increased *LOC100294145* expression as compared to the intermediate genotype “GT” (*p* < 0.05) and the major genotype “GG” (*p* < 0.01) (Fig. 2A). For rs115388857, an eQTL dosage effect of increasing *LOC100294145* expression was observed in the minor genotype “GG” compared to the intermediate genotype “CG” (*p* < 0.001) and the major genotype “CC” (*p* < 0.0001) (Fig. 2B). *LOC100294145* expression was significantly higher in AD subjects as compared to atopic non-AD subjects (*p* < 0.05; Fig. 2C) and non-atopic non-AD subjects (*p* < 0.01; Fig. 2C). This suggests that the risk allele “T” of rs116160149 increases AD susceptibility by increasing *LOC100294145* expression.

### Promoter activity of *LOC100294145* is regulated by rs116160149 and rs115388857

**Fig. 3:**
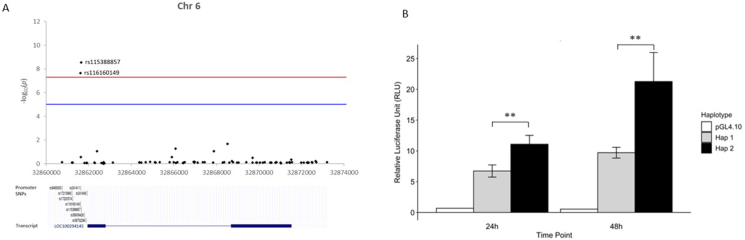
(A) The Manhattan plot (top) shows the genotype-phenotype association for SNPs in the 2 kb upstream region of *LOC100294145*. The red and blue lines represents the GWAS significant (*p* < 5 × 10^−8^) and GWAS suggestive threshold (*p* < 1 × 10^−5^) respectively. SNPs rs116160149 and rs115388857 are highlighted as shown. Positions of the promoter SNPs relative to the *LOC100294145* gene are shown (bottom). (B) Relative luciferase units (RLU) of haplotype 1 and haplotype 2 were compared to investigate the effect of risk alleles on *LOC100294145* promoter activity (∗∗*p* < 0.01). The RLU were measured 24 and 48 h in HEK293 cells post-transfection. The control is a promoter-less pGL4.10

The SNPs rs116160149 (minor allele = “T"; *p* = 2.175 × 10^−8^; OR = 1.552) and rs115388857 (minor allele = “G"; *p* = 2.81 × 10^−9^; OR = 1.55) were the only SNPs ±2 kb of *LOC1*00294145 that passed the GWAS significant threshold of 5 × 10^−8^ (Fig. 3A). The relative positions and genotype-phenotype association of all SNPs in the ±2 kb region of *LOC100294145* are shown in Fig. 3A.

Common promoter haplotypes (frequency >1%) carrying both the major alleles (“GC”) and minor alleles (“TG”) of rs116160149 and rs115388857 respectively were identified in our genetic association cohort. An *in* *vitro* luciferase assay was thus performed to investigate if both rs116160149 and rs115388857 are functional SNPs that regulate *LOC100294145* promoter activity in tandem. The promoter region of *LOC100294145* carrying either 1 of the 2 identified haplotypes was cloned 2 kb upstream of a luciferase reporter gene. Haplotype 1 carries protective alleles (“GC”) and haplotype 2 carries risk alleles (“TG”) of rs116160149 and rs115388857 respectively. A significantly higher level of expression in haplotype 2 was observed as compared to haplotype 1 24- (*p* < 0.01) and 48- (*p* < 0.01) hours post-transfection (Fig. 3B), supporting the hypothesis that the risk alleles of rs116160149 and rs115388857 significantly increase the promoter activity of *LOC100294145*. This further validates that rs116160149 and rs115388857 mediates AD risk through regulating *LOC100294145* expression.

### Co-expression analysis suggests involvement of *LOC100294145* in interleukin-27 and type 1 interferon signaling

**Fig. S1:**
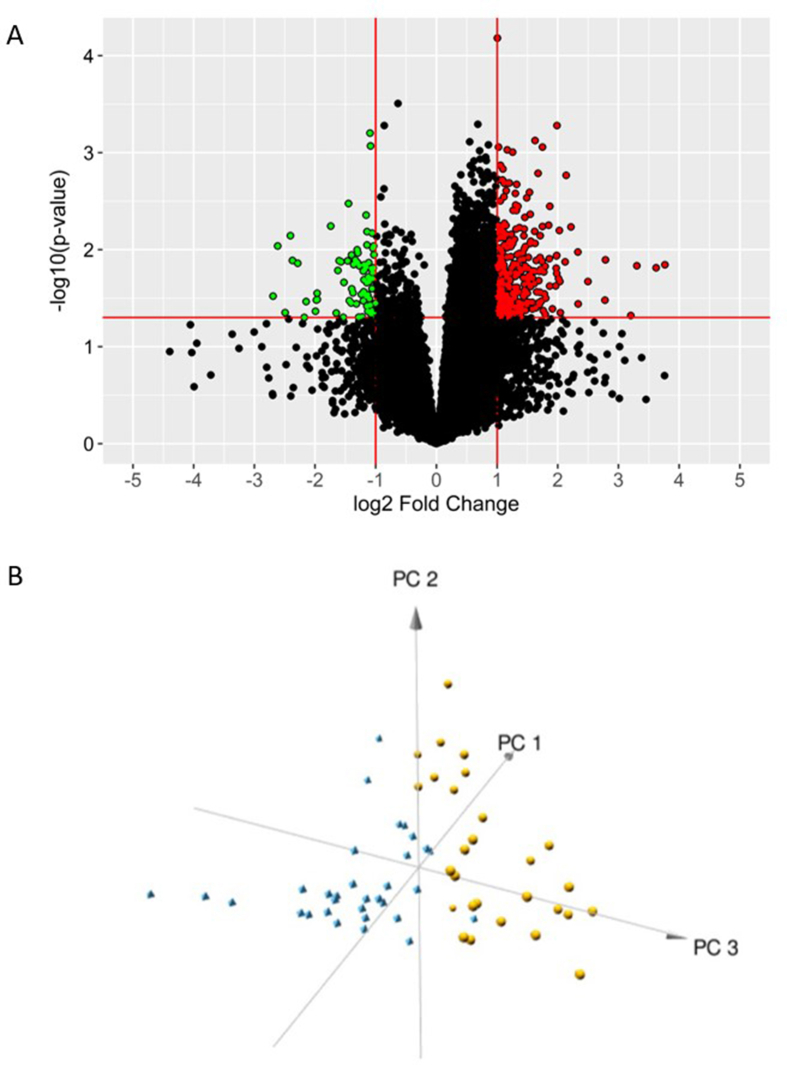
(A) Volcano plot of differentially co-expressed genes (p-value <0.05 and |log-2 fold change| > 1). Upregulated and downregulated genes are shown in red and green respectively. (C) PCA clustering using differentially co-expressed genes (p-value <0.05 and |log-2 fold change| > 1). AD and healthy subjects are represented by the yellow and blue points respectively

**Fig S2:**
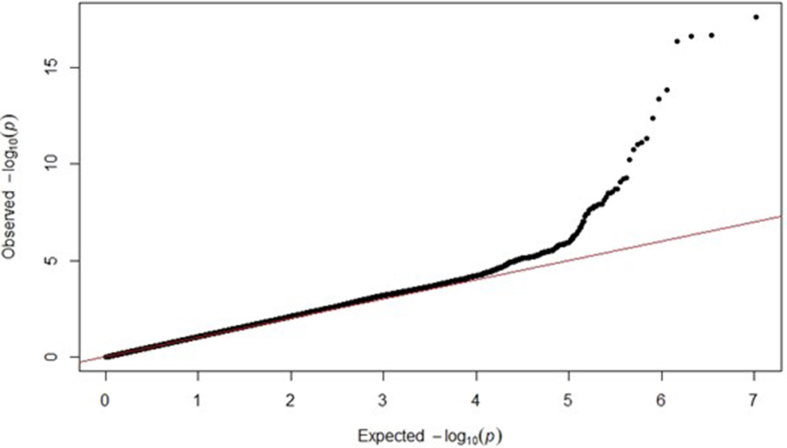
Quantile-Quantile plot of the observed against expected p-values in negative logarithmic scale. The red dots represent the p-value of all SNPs in the genome wide association study of atopic dermatitis patients versus non-atopic controls. The black diagonal line represents the expected p-values in a normal distribution. A genomic inflation factor of close to 1 implies no evidence of inflation. Genomic inflation factor: 1.086

**Fig. S3:**
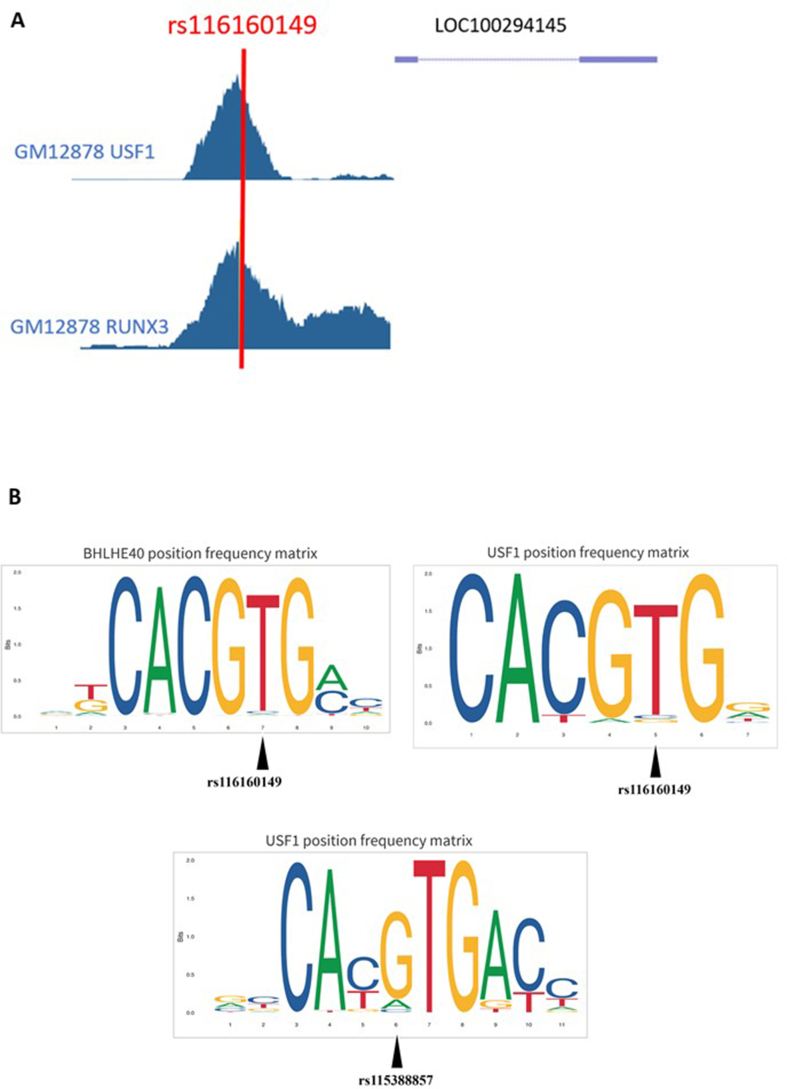
Predicted transcription factors overlapping SNPs rs116160149 and rs115388857. (A) ChIP-seq peaks of the transcription factors USF1 and RUNX3 on the promoter of LOC100294145 in the GM12878 lymphoblastoid cell line, at a genomic region overlapping AD-associated SNPs rs116160149. The ChIP-seq data is retrieved from RegulomeDB. (B) Sequence logo diagram retrieved from JASPER (https://jaspar.genereg.net/) showing frequency matrix profiles of BHLHE40 and USF1 transcription factor binding motifs. The letters represent the possible bases at respective positions in the motif, where the height of the letter indicates the frequency of the nucleotide observed in that position. Variants with higher nucleotide frequency increases the motif score and hence the likelihood of the transcription factor binding to the site. Black arrow indicates position of respective SNP of interest

Researchers commonly employ co-expression studies to predict the functions of non-coding RNAs, leveraging the "guilt-by-association" principle. This principle posits that genes with similar expression patterns are likely to share analogous functions.^47^^,^^48^ Consequently, genes regulated by a long non-coding RNA (lncRNA), such as *LOC100294145*, are expected to exhibit correlated expression with the lncRNA itself.^47^ The elucidation of the lncRNA's function is then achieved by analysing the enriched pathways of co-expressed genes, applying the "guilt-by-association" principle. In a study by Schwarzer et al, for example, co-expression predicted LINC00173 to control myeloid differentiation, and this was subsequently validated in a knockdown experiment.^49^ We thus investigated a list of co-expressed genes whose expressions were significantly correlated with that of *LOC100294145*. These genes were also tested to be differentially expressed between AD patients and healthy controls. A diagnostic PCA plot shows a distinct expression profile between AD cases and non-AD non-atopic controls (*p* < 0.05, |log2 fold change| > 1) in the functional association cohort, suggesting that the AD phenotypes are well-separated (Figure S1). *LOC100294145* was then functionally annotated based on the enrichment in biological functions of its co-expressed genes.

**Fig. S4:**
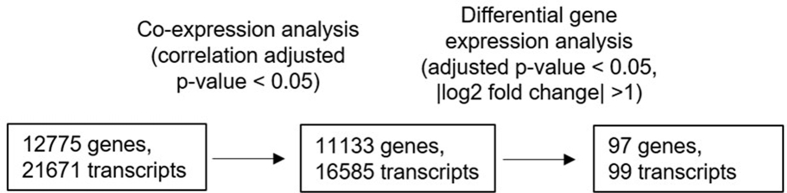
Flowchart documenting the steps and results of the co-expression analysis

Whole transcriptome co-expression analysis was performed using PBMCs in the functional association cohort (Figure S4). Out of 12 775 known expressed coding genes and 21 671 isoforms, 11 133 genes and 16 585 isoforms were identified as co-expressed genes whose expressions were significantly correlated with that of *LOC100294145* (FDR adjusted *p* < 0.05). Of these, the expressions of 97 genes and 99 isoforms were also significantly associated with AD (FDR adjusted *p* < 0.05) with a log2 fold change more than 1 (Table S5). Gene enrichment analysis also showed that these genes are involved in various inflammatory processes including interleukin-27 and type 1 interferon signaling (Table S6).

### Hub genes of AD co-expression network are also enriched in interleukin-27 and type 1 interferon signaling

**Fig. 4:**
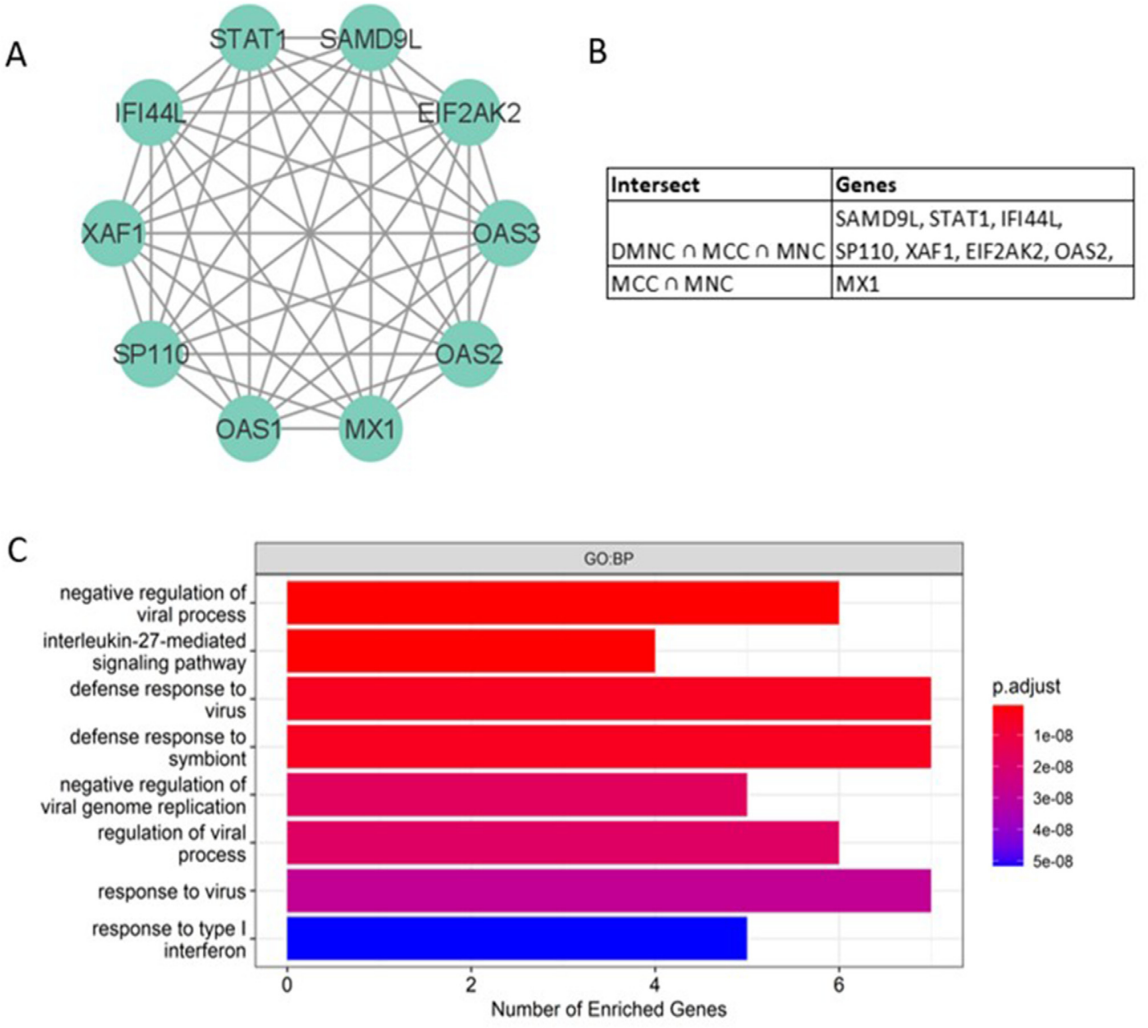

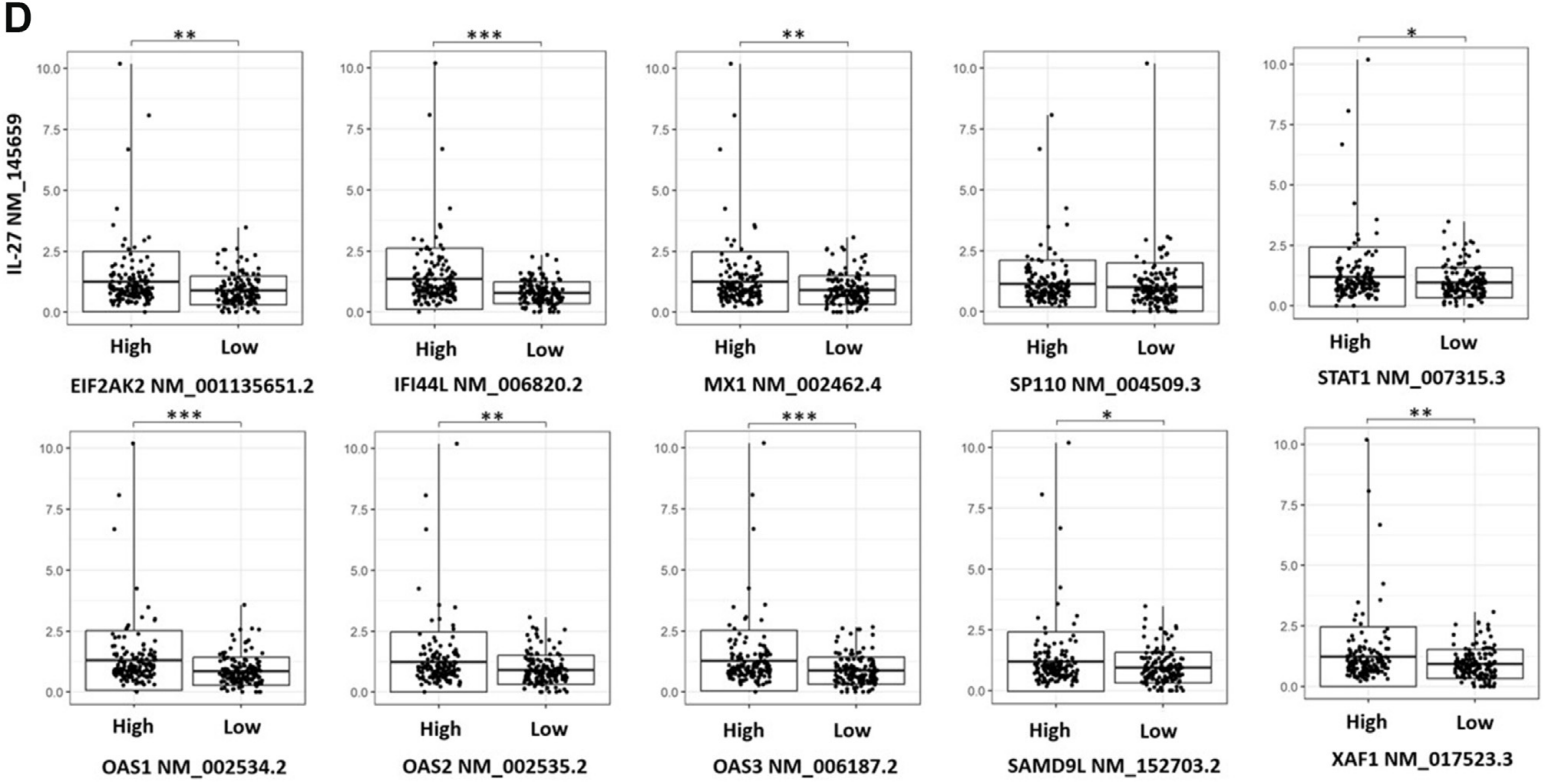

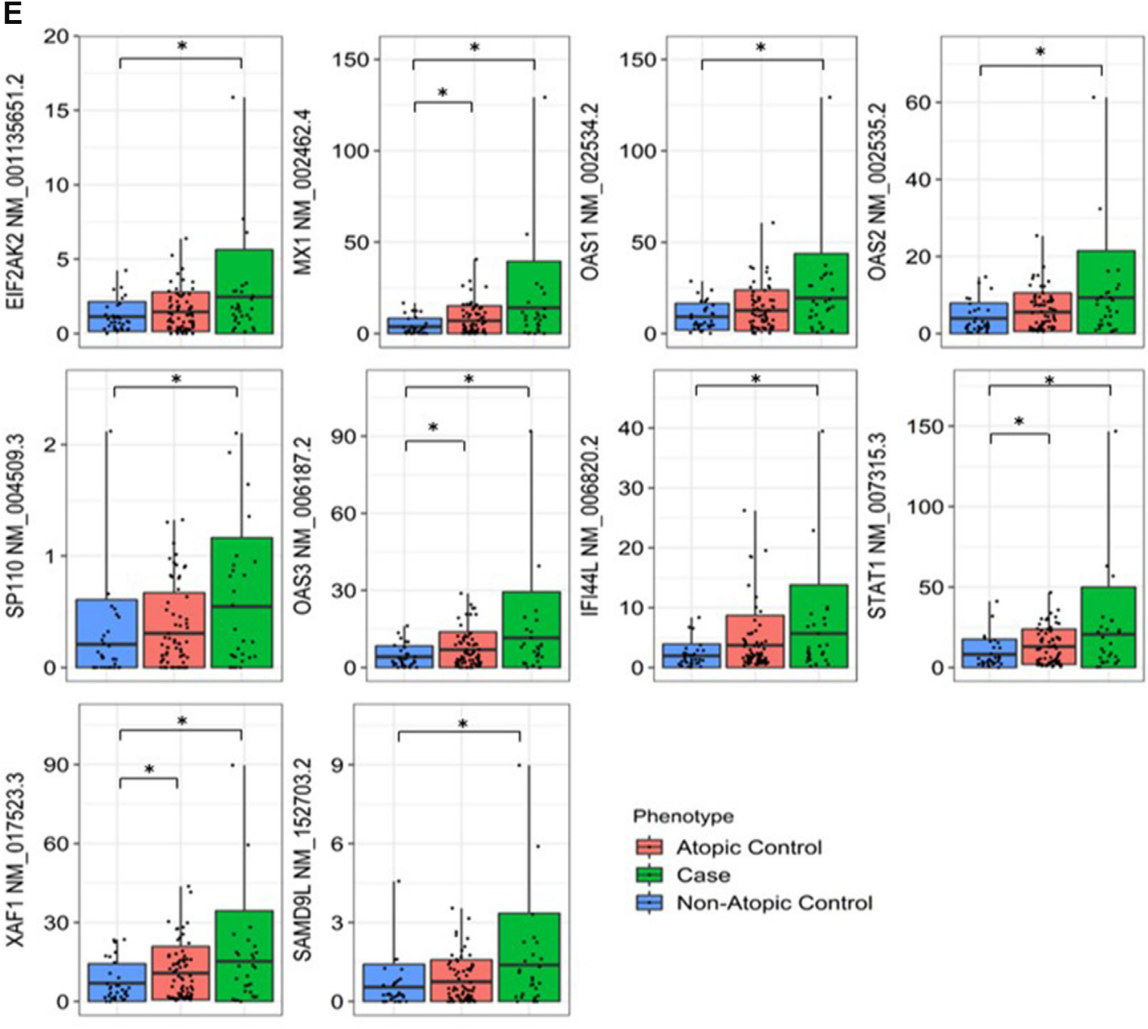
(A) Co-expression PPI network identified based on the clustering of significantly interacting proteins using MCODE. (B) Hub genes that are important for biological functions identified, based on their connectivity in the protein-protein interaction network. The genes that intersect at least 2 analyses (*MCC, DMNC, MNC*) in Cytohubba are defined as hub genes. (C) Functional analyses of hub genes by g:Profiler using the GO:BP database. Pathways enriched for the hub genes are shown. The y-axis shows the enriched pathway and the x-axis shows the number of enriched genes. The adjusted p-value is shown on a colour scale. (D) Whole blood PBMCs positive association showing upregulation of *IL-27* with higher levels of 9 out of 10 hub genes expression (split by median) in the functional association cohort (∗∗*p* < 0.01,∗∗∗*p* < 0.001). (E) Differential gene expression analyses showing the association between AD phenotypes and expression of hub genes in the functional association cohort (∗*p* < 0.05)

Hub genes are defined as genes that are highly connected in protein-protein interaction (PPI) networks. These genes perform critical biological functions within the PPI network and are significantly associated with disease status as compared to genes with few interaction partners.^50^ Since studies have suggested disease genes to have a higher number of interaction partners as compared to non-disease genes,^51^ we constructed a protein-protein interaction (PPI) network consisting of 97 proteins and 89 interactions with the differentially co-expressed genes. Analyses with MCODE identified a significantly interacting subnetwork consisting of 10 proteins (*EIF2AK2*, *IFI44L*, *MX1*, *OAS1*, *OAS2*, *OAS3*, *SAMD9L*, *SP110*, *STAT1*, *XAF1*) and 45 interactions, with a network score of 10 (Fig. 4A). These 10 proteins were also identified as hub genes in an independent analysis using Cytohubba (Fig. 4B–Table S7), where highly connected hub genes have been shown to be functionally important in the manifestation of diseases.^28^^,^^52^, ^53^, ^54^, ^55^ Of the 10 identified hub genes, 5 are involved in type 1 IFN signaling (*p* = 5.16 × 10^−8^) and 4 are involved in interleukin-27-mediated signaling pathway (*p* = 6.36 × 10^−10^) (Fig. 4C). Analysis of IL-27 to hub genes transcript expression levels in PBMCs also showed a strong significant positive association with 9 out of the 10 identified hub genes (Fig. 4D). The identified hub genes are also implicated in immune-related pathology (Table S8). These 10 genes are also all significantly higher in AD cases as compared to non-atopic non-AD controls in PBMCs of our functional association cohort (Fig. 4E).

### *LOC100294145* could regulate identified hub genes

**Fig. 5:**
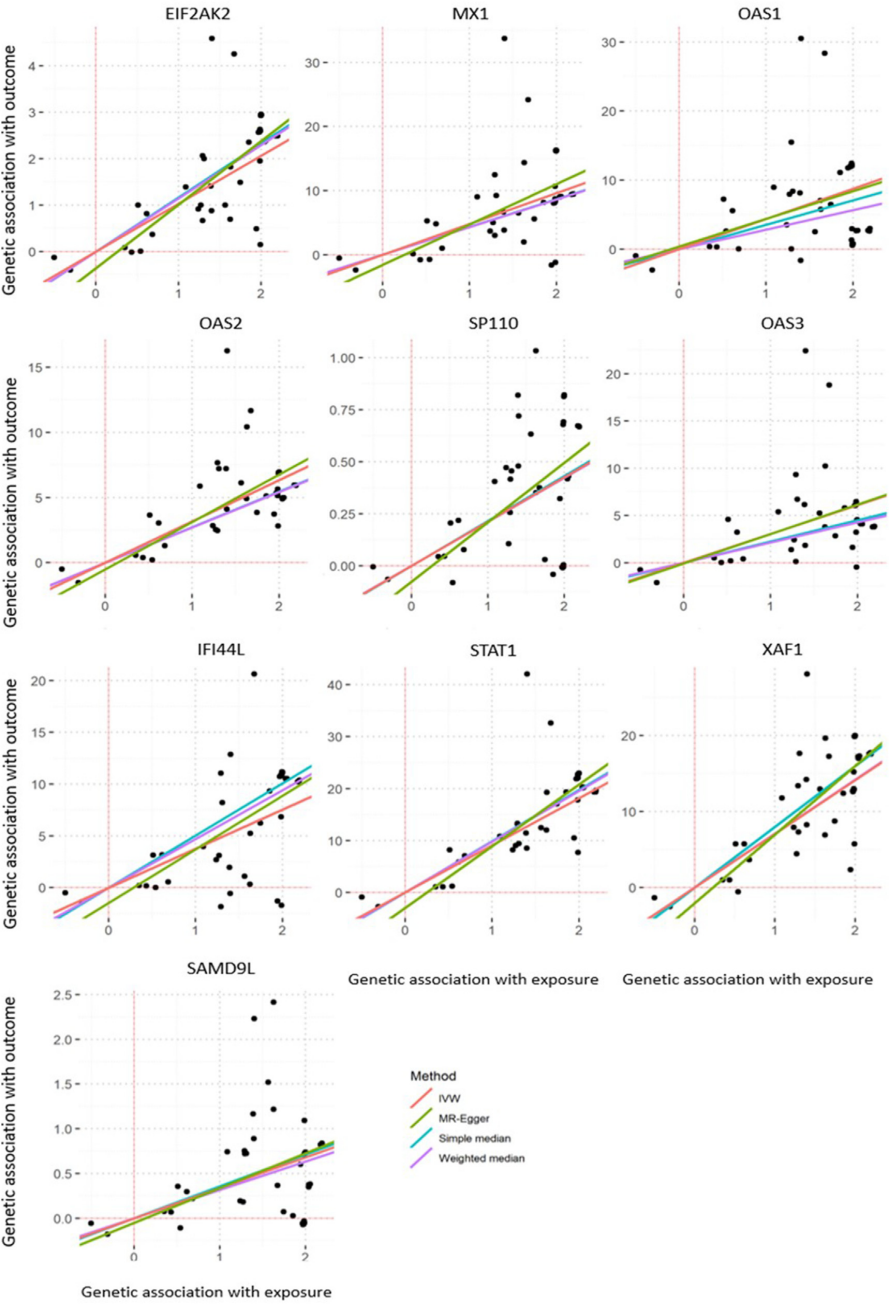
Scatter plot for the MR causal estimates of *LOC100294145* on the expression of each hub gene. The methods used are: inverse variance weighted, simple median, weighted median and MR-Egger. The slope of each line represents the causal estimated using each method

**Fig. S5:**
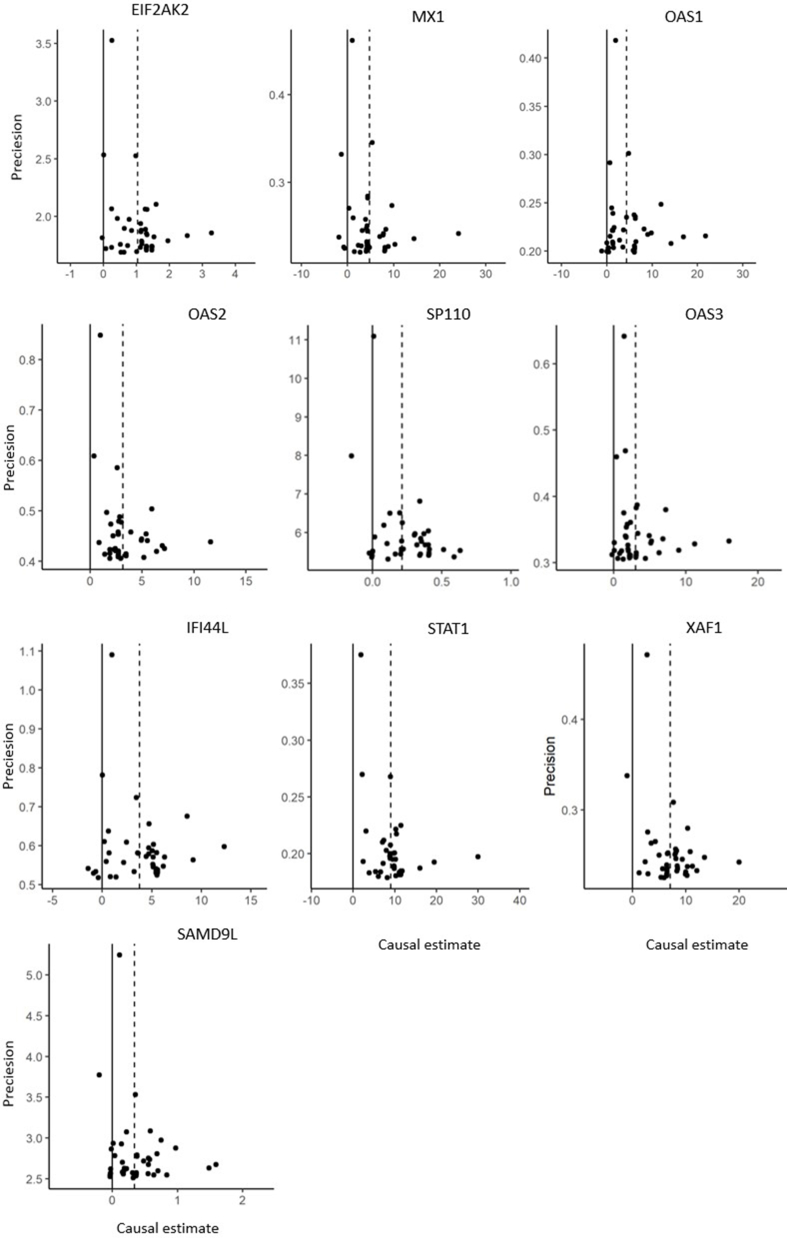
Funnel plot for the MR analyses of the causal effect of LOC100294145 on the expression of each hub gene, for a visual assessment of heterogeneity. The plot is narrow at the top and wide at the bottom. The funnel plot shows the precision of the instrumental SNPs against the causal estimate

Long non-coding RNAs were found to regulate gene expression through a myriad of mechanisms such as chromatin remodeling, alteration of mRNA translation and stability, as well as signaling pathway interference.^56^, ^57^, ^58^ They have also been implicated in complex diseases due to their regulatory functions.^59^ To investigate the regulatory role of *LOC100294145* on the hub genes, MR was performed using *LOC100294145* expression as the exposure and the expression of all 10 hub genes as the outcome. We identified 45 SNPs that were independently associated with *LOC100294145* expression from the functional association cohort as instrument SNPs (*p* < 1 × 10^−5^, LD < 0.1). These SNPs had F-statistics more than 10, suggesting that they are strong instruments. In the preliminary MR analyses using IVW, the causal estimate plots and diagnostic funnel plot of the MR analysis on each *LOC100294145*-hub gene pair is shown in Fig. 5 and figure S5. The IVW analyses suggested a regulatory role of *LOC100294145* on all transcripts where there is a positive and significant causal association (*p* < 0.05; Fig. 5, Table S9). The causal estimates stand for the increase in outcome given 1 standard deviation increase in exposure. In this study, that would mean that a standard deviation increase of *LOC100294145* expression results in an increase in MX1 expression by 4.81 units.

Sensitivity analyses were also performed using 3 other MR methods, namely: simple median, weighted median and MR egger. All methods yielded similar causal estimates for the 10 genes (Fig. 5, Table S9). We found that 5 out of the 10 genes (*MX1, OAS1, OAS2, OAS3, SAMD9L*) did not show significant evidence of horizontal pleiotropy (IVW Cochran Q *p* > 0.05, MR-Egger intercept *p* > 0.05, MR-Egger Cochran Q *p* > 0.05), indicating that they are causally regulated by *LOC100294145*. The other 5 genes (*EIF2AK2, SP110, IFI44L, STAT1, XAF1*) on the other hand, exhibited the presence of horizontal pleiotropy and their causal association should be interpreted with caution (IVW Cochran Q *p* < 0.05, MR-Egger intercept *p* < 0.05, MR-Egger Cochran Q *p* < 0.05). Lastly, the MR-PRESSO analysis detected outlier instruments when investigating the causal relationship of *LOC100294145* to *IFI44L* (Table S9). Pleiotropy was still evident following the removal of outlier SNPs.

### Hub genes are differentially expressed between AD phenotypes in independent cohorts and downregulated following NB-UVB treatment

**Fig. 6:**
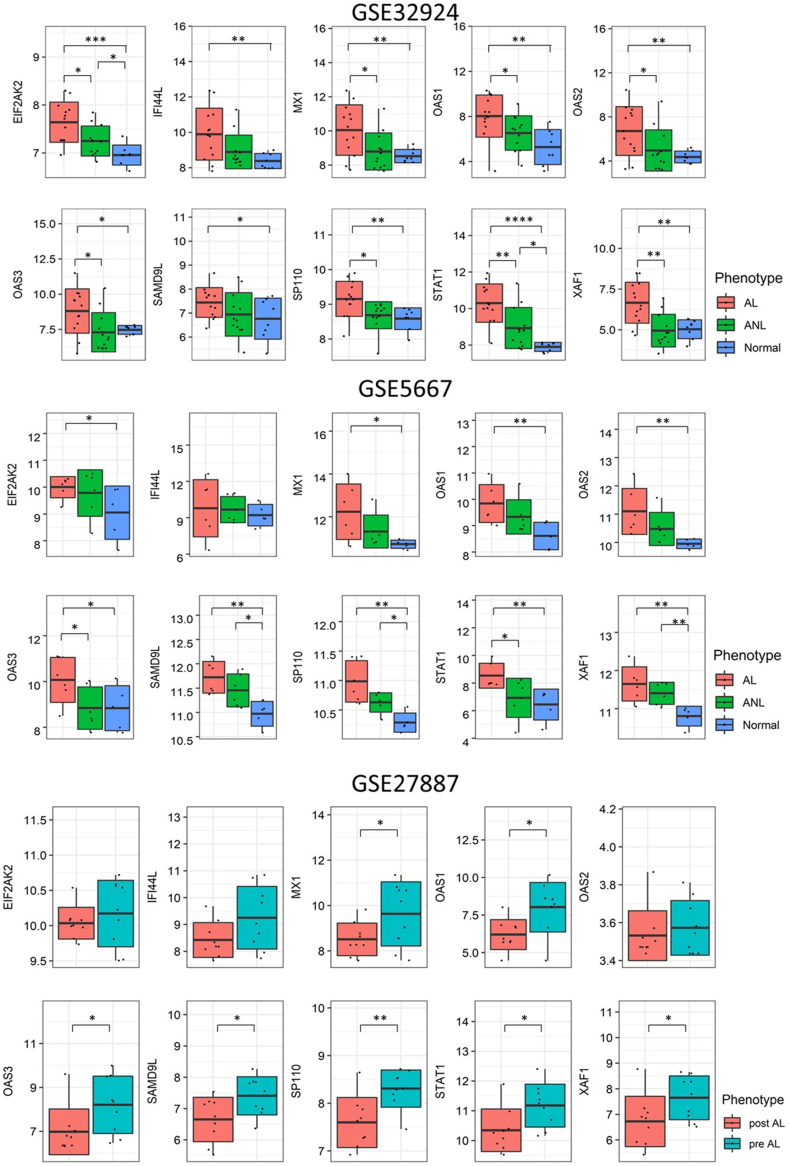
The comparison of the expression of hub genes between non-lesional AD, lesional AD, and healthy skin are shown in GSE5667 and GSE32924. The comparison of the expression of hub genes between AD skin pre- and post-NB-UVB therapy is shown in GSE27887. All GEO datasets measured gene-level expressions (∗*p* < 0.05, ∗∗*p* < 0.01, ∗∗∗*p* < 0.001, ∗∗∗∗*p* < 0.0001)

Four expression datasets (GSE5667, GSE32924, GSE27887, GSE32473) were downloaded from the GEO database to study the association of hub genes to AD phenotypes and treatment regimens.^31^, ^32^, ^33^, ^34^, ^35^, ^36^ In GSE5667, we observed that all hub genes, except for *IFI44L*, were upregulated in skin biopsies of lesional AD skin compared to in normal skin (Fig. 6). In GSE32924, all 10 hub genes were upregulated in lesional AD skin compared to in normal skin (Fig. 6). These findings suggest that interleukin 27 and type 1 interferon signaling may be dysregulated to different extents in AD lesional and non-lesional or normal skin.

**Fig. S6:**
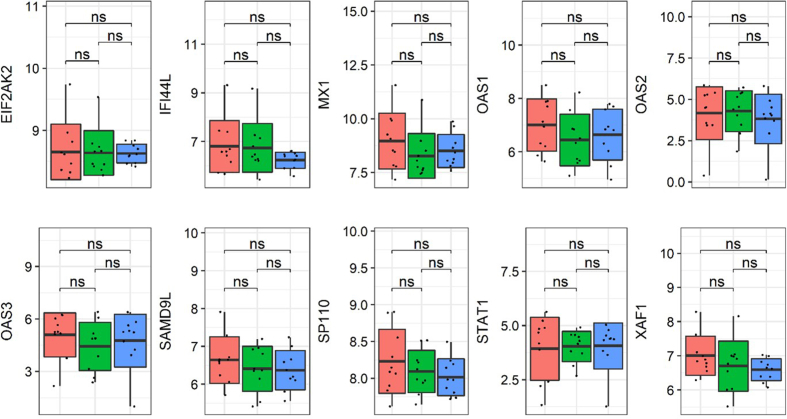
Expression of hub genes post-betamethasone and pimecrolimus treatment is shown (GSE32473). The expression of hub genes remain unchanged

In addition, a study (GSE27887) performed using narrow-band ultraviolet-B (NB-UVB) therapy resulted in a significant decrease in 7 (*SAMD9L*, *STAT1*, *SP110*, *XAF1*, *OAS1*, *OAS3*, *MAX1*) out of 10 hub genes' expression post-treatment (Fig. 6). On the other hand, in GSE32473 dataset involving treatment with betamethasone and pimecrolimus, no significant changes to hub genes’ skin expression levels were observed (Figure S6). Clinically, this suggests that AD patients with interleukin 27 and type 1 IFN-related pathogenesis may be responsive to NB-UVB but not to topical treatments.

### Autoallergic propensity with higher *LOC100294145* expression

Type 1 IFNs form the central elements of several systemic autoimmune diseases. We thus evaluated the auto-allergic responses of 111 participants from the functional association cohort. Individuals were grouped based on *LOC100294145* transcript expression in PBMCs split by median. Presence of autoallergy is determined by individuals having a serum IgE titer greater than 3.5 kU/L (Class 3 and above) to the tested human fungal homolog allergens. In those presenting with higher *LOC100294145* expression, 6.8% (3/44) were autoallergic to human enolase (GenBank ID: NM_001428.5), 4.5% (2/44) to human heat shock protein (GenBank ID: NM_006597.6) and 4.5% (2/44) to human aldehyde dehydrogenase (GenBank ID: AAA51693.1) as compared to the 1.5% (1/67) autoallergy to all 3 of these fungal homologs in those with lower *LOC100294145* expression. Autoallergy to Human 60S acidic ribosomal protein P2 (GenBank ID: AJ224333) was also greater at 13.6% (6/44) in those with higher *LOC100294145* expression in comparison to the 7.5% (5/67) in those with lower *LOC100294145* expression. Taking into consideration all the human autoallergens tested, 18.2% (8/44) of individuals with high *LOC100294145* expression had serum IgE titers greater than 3.5 kU/L (Class 3 and above) to at least 1 of the 7 tested human fungal homolog allergens as compared to the 9% (6/67) with lower *LOC100294145* expression (Fisher's exact test *p* = 0.2415). This suggests that higher *LOC100294145* expression could increase the propensity of autoallergic responses.

## Discussion

This study has attempted to characterize the function of a previously unknown lncRNA *LOC100294145* in the context of AD. The identification of *LOC100294145* using the triangle method proposes that when multiple levels of omics data point to the same association, the identified gene is more likely to be a true positive since it eliminates inconsistent information from each data type.^13^ Disease association is also strengthened by the enrichment of the identified trait-associated SNPs in eQTLs and predictions of regulatory roles.^40^ We also conducted an *in* *vitro* luciferase promoter assay validating the effect of rs116160149 and rs115388857 in influencing *LOC100294145* promoter activity. Functional annotations of TFBS and PPI in the co-expression network also further supports our hypothesis. Given that the study of genetic risk in complex diseases such as AD is often plagued with the problem of missing heritability, with less than 20% of AD heritability explained using GWAS,^10^ such multi-stage approach can therefore serve as an effective strategy to bridge that missing heritability problem. It allows for the refining and narrowing of the dataset at each step, alleviating obstacles surrounding multiple testing.^13^ The application of biological constraints, such as promoter binding on GWAS SNPs, have also enhanced identification of true positive hits.^60^

**Fig. S7:**
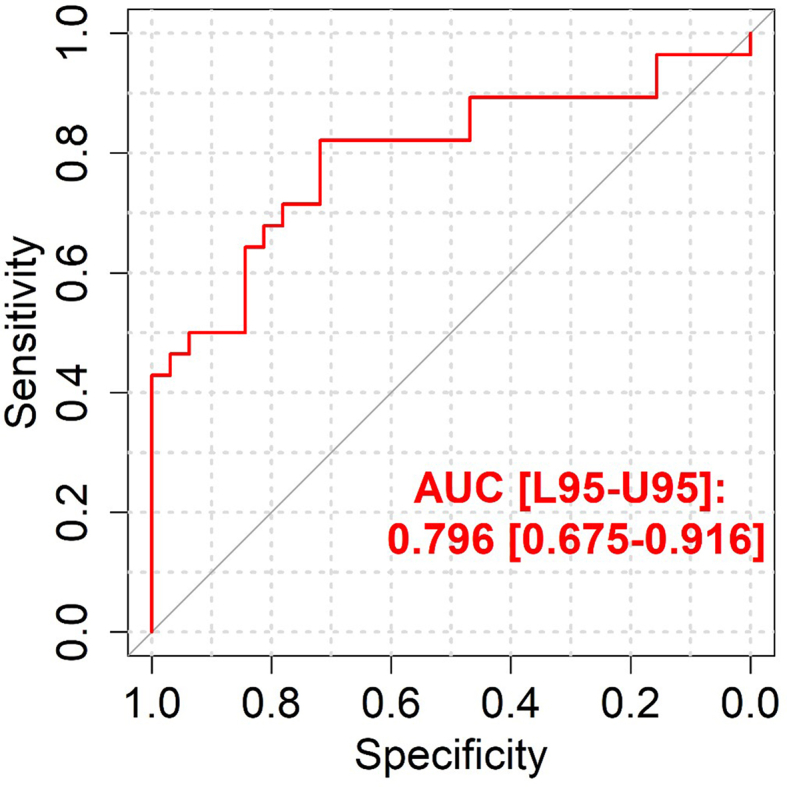
ROC AUC of the combined expression of all hub genes against the AD phenotype, demonstrating the diagnostic value of the combined expression of hub genes in discriminating AD phenotypes

The correlative co-expression studies serve as an initiatory step, providing confidence and direction to elucidating the functional impact of novel genetic variants, increasing the power of uncovering the genetic architecture of complex diseases such as AD. LncRNAs are key regulators of inflammation and have been implicated in AD pathogenesis in other studies.^61^, ^62^, ^63^, ^64^ Through the “guilt-by-association” principle in a co-expression study, we elucidated that *LOC100294145* is involved in interleukin-27 and type 1 interferon signaling. Future studies involving molecular perturbations can then be used to further validate our finding that *LOC100294145* causally regulates the hub genes enriched in IL-27 and type 1 IFN signaling. In the functional association cohort, the combined expression of the 10 hub genes provided a good diagnostic value for AD, with a receiver operating characteristic area under the curve (ROC AUC) of 0.796 (Figure S7). This suggests the potential use of these genes to identify subgroups of AD patients with dysregulated interleukin-27 and type 1 interferon signaling, aiding in the prediction of treatment efficacy.

In type 1 IFN signaling, T cell activation is enhanced due to the increased expression of co-stimulatory molecules, MHC class I molecules and MHC class II molecules on dendritic cells (DCs).^65^^,^^66^ In agreement, the expression of MHC class II and costimulatory molecules were increased in Langerhans cells in a mouse model of AD development.^67^ T cell survival and the activity of cytotoxic lymphocytes are also enhanced due to type 1 IFN signaling, and a study found that autoreactive cytotoxic CD8 T cell numbers were elevated in AD patients upon induction by the autoallergen hom s 2.^68^, ^69^, ^70^, ^71^ Together, the autoallergens released during tissue damage due to excessive cytotoxic T cell activities and enhanced antigen presentation can elicit the autoallergy observed in AD.^72^, ^73^, ^74^, ^75^, ^76^ From our own serum data, the presence of autoallergy following upregulation of *LOC100294145* was also suggested. In those presenting with higher *LOC100294145* expression, 18% had IgE titers greater than 3.5 kU/L (Class 3 and above) to at least 1 of the 7 tested human fungal homolog autoallergens. This is 2 times higher than the 9% of individuals with lower *LOC100294145* expression. The preliminary data obtained is suggestive of a positive association between higher levels of *LOC100294145* expression and an increased risk of autoallergy. This can be further evaluated with a larger cohort size.

Type I IFN also upregulates the expression of IL-22R, which is produced exclusively by keratinocytes in the skin.^77^ Type 1 IFN signaling is associated with increased Th17 polarization in autoimmune diseases like psoriasis and systemic lupus erythematosus, resulting in the production of IL-22 by Th17 cells.^78^ The presence of the autoallergen Hom s 2 also induces IL-17 and IL-22 by PBMCs.^79^ In combination, sustained IL-22 signaling results in epidermal hyperplasia due to keratinocyte hyperproliferation.^80^ Interestingly, higher Th17 T-cell responses are also associated with greater epidermal hyperplasia in individuals with AD.^81^^,^^82^ As such, we propose type 1 interferon signaling contributes to AD pathogenesis by driving epidermal hyperplasia and autoallergy.

**Fig. 7:**
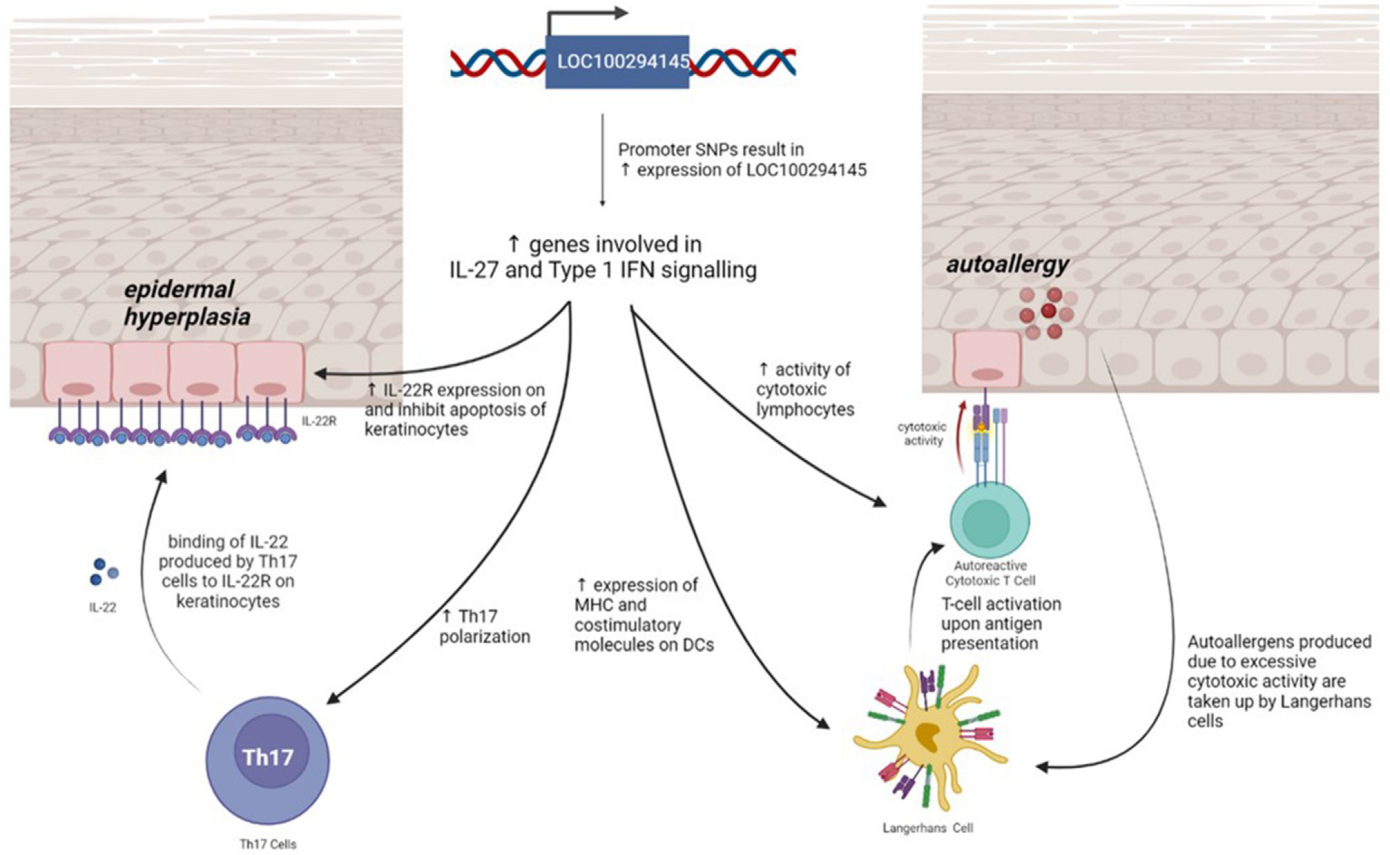
Proposed mechanisms of *IL-27* and type 1 IFN signaling in AD pathogenesis. The increased expression of *LOC100294145* due to the risk alleles of rs116160149 and rs115388857 mediates increased *IL-27* and type 1 IFN signaling, which contributes to the development of AD through epidermal hyperplasia and autoallergy. Figure created with Biorender

The function of IL-27 is closely related to that of type 1 IFNs. Antigen-presenting cells produce IL-27 in early immune responses, which was found to induce CXCL10 upregulation in keratinocytes and similarly increase MHC class 1 production in eczematous skin.^83^ CXCL10 chemokine attracts Th1 cells, resulting in increased activation and maturation of antigen-presenting cells, promoting inflammatory responses.^84^ IL-27 was also found to activate CD8 T cells^85^ and inhibit apoptosis of keratinocytes, allowing continued CXCL10 production and inflammatory responses.^83^ Studies have also demonstrated a bi-directional relationship between Type 1 IFNs and IL-27, where each molecule can induce the expression of the other.^86^ As such, both IL-27 and type 1 IFN signaling contributes to AD pathogenesis in tandem. The elucidated AD pathogenesis is illustrated in Fig. 7.

Analyzing independent gene expression data, we found that the hub genes enriched in type 1 IFN signaling were not only differentially expressed between healthy and AD lesional skin, but also between non-lesional and lesional AD skin (GSE5667, GSE32924).^31^, ^32^, ^33^, ^34^ This suggests the involvement of IL-27 and type 1 IFN signaling in AD. Our observed whole blood significant positive association between elevated *IL-27* and high levels of hub genes expression further supports this proposition (Fig. 4D). Furthermore, hub genes enriched in type 1 IFN signaling were downregulated following NB-UVB treatment for AD (GSE27887).^35^ The study also reported a significant correlation of CD8 T cell markers and *IL-22* expression with AD disease severity as well as their decrease in expression post-treatment.^35^ This further supports our hypothesis that identified mechanisms of type 1 10.13039/501100007072IFN signaling and IL-27 contribute to AD through IL-22 signaling and CD8 T cell activation (Fig. 7).

**Fig. S8:**
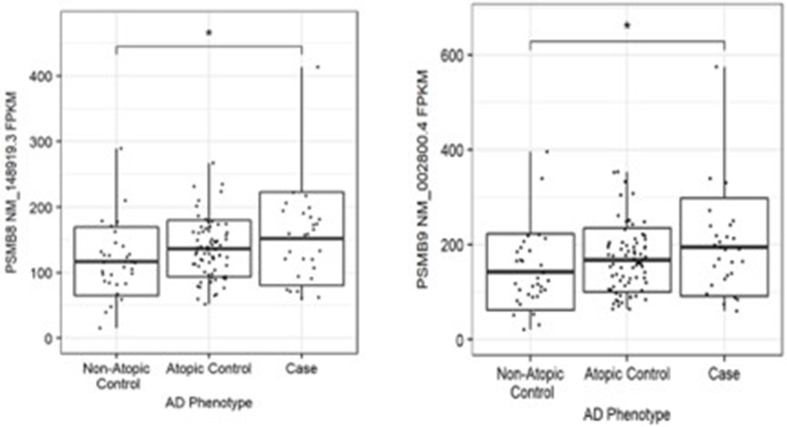
Differential gene expression analyses showing the association of AD phenotypes with PSMB8 and PSMB9 expression in the functional association cohort (∗p < 0.05).

Other studies have also reported similar elevation in Th17 and CD8 T cells infiltration with the presentation of clinical lesions.^87^^,^^88^ In our functional association cohort, we also found type 1 IFN-induced immunoproteasome subunits (*PSMB8* and *PSMB9*) essential for antigen presentation and CD8 T cell activation to be upregulated in AD cases (Figure S8).^89^, ^90^, ^91^ Since AD is a heterogenous disease that consists of multiple endophenotypes and a “one-size-fits-all” approach is insufficient to maximize treatment efficacy,^92^, ^93^, ^94^ this makes the type 1 IFN signaling pathway an attractive target to achieve a more effective and personalized treatment for AD patients with a dysregulated type 1 IFN signaling profile.

**Fig. S9:**
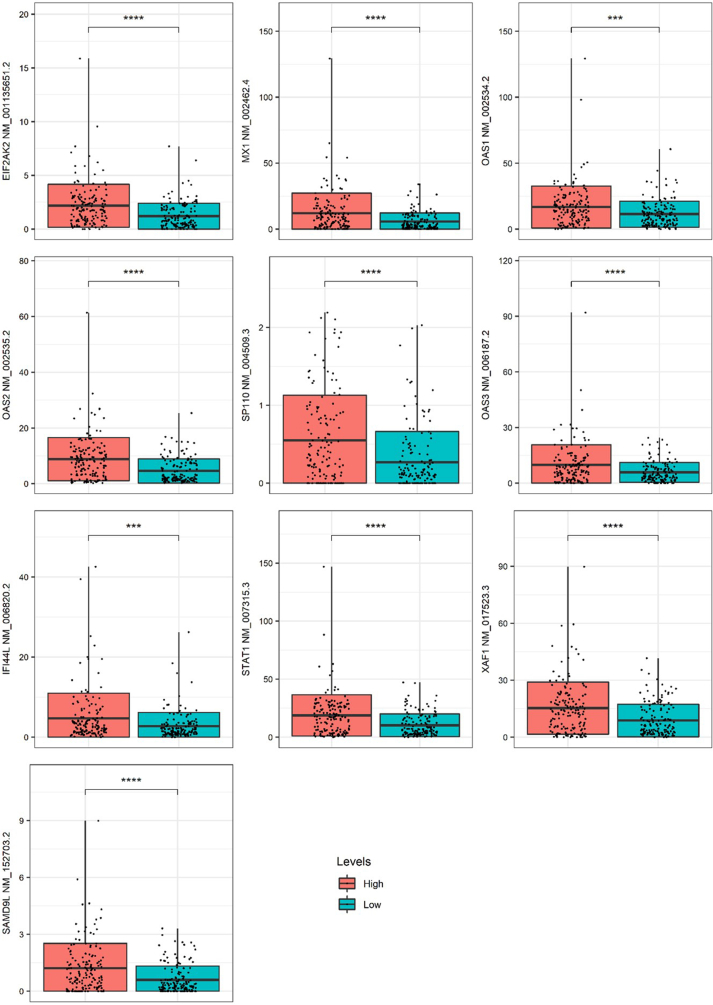
Upregulation of hub genes in individuals with higher levels of inflammation in the functional association cohort (∗∗p < 0.01,∗∗∗p < 0.001,∗∗∗∗p < 0.0001).

In another study by Jensen et al, although betamethasone and pimecrolimus treatment were found to resolve inflammation in lesional AD skin, no significant change in hub genes expression levels were observed post-treatment (GSE32473).^36^ This implies that for AD patients with a dysregulated type 1 IFN signaling profile such topical treatment methods may not be maximally effective. Adverse effects could even result from long-term use.^95^^,^^96^ *LOC100294145* and hub genes expression levels could therefore serve as markers for predicting treatment efficacy. Furthermore, therapeutics targeting the type 1 IFN signaling pathway provides a feasible alternative as the downregulation of pathway hub genes is associated not only with reduced inflammation as observed in the functional associated cohort (Figure S9), but is also observed with the reversal of epidermal pathology in a study by Tintle et al (GSE27887).^35^ Studies have also shown lncRNA therapies to have fewer side effects with high specificity, and the multi-level gene regulation by lncRNA makes it an attractive pharmacological target.^56^, ^57^, ^58^^,^^97^

In summary, our study identified the minor alleles of rs116160149 and rs115388857 in *LOC100294145* gene promoter to increase AD risk by 1.552 (*p* = 2.18 × 10^−8^) and 1.55 (*p* = 2.81 × 10^−9^) -fold respectively via the upregulation of *LOC100294145* expression. Using the multi-omics analyses approach we provided evidence that this identified risk locus is a true causal variant influencing *LOC100294145* expression and potentially contributes to AD pathogenesis by regulating hub genes involved in type 1 IFN signaling. Our hypothesized downstream pathway was also supported in parallel with other reported independent gene expression datasets. This study therefore highlights the efficacy of a multi-omics approach in identifying and functionally characterizing novel genetic risk factors for complex diseases, even for those with small effect sizes such as in the case of *LOC100294145*. Success in lncRNA therapy for cancer has already been reported^98^ and our identification of *LOC100294145* could pave the way for the development of more personalized therapies such as those targeting dysregulated *LOC100294145* expression.

## Abbreviations

AD, Atopic dermatitis; CI, confidence interval; DCs, dendritic cells; DEGs, differentially expressed genes; eQTL, expression quantitative trait loci; GEO datasets, gene expression omnibus datasets; GO:BP, Gene Ontology: Biological Processes; GWAS, genome wide association studies; IFN, interferon; ISAAC, International Study of Asthma and Allergies in Childhood; KEGG, Kyoto Encyclopedia of Genes and Genomes; LD, linkage disequilibrium; lncRNA, long non-coding RNA; MCODE, Molecular Complex Detection; MR, mendelian randomization; NB-UVB, narrow-band ultraviolet B; OR, odds ratio; PBMCs, peripheral blood mononuclear cells; PPI, protein-protein interaction; ROC AUC, receiver operating characteristic area under the curve; SNPs, single nucleotide polymorphisms; TFBS, transcription factor binding sites.

## Funding

F.T.C. has received research support from the National University of Singapore, Singapore Ministry of Education Academic Research Fund, Singapore Immunology Network (SIgN), National Medical Research Council (NMRC) (Singapore), Biomedical Research Council (BMRC) (Singapore), National Research Foundation (NRF) (Singapore), Singapore Food Agency (SFA) (Singapore), and the Agency for Science Technology and Research (A∗STAR) (Singapore); Grant Numbers are N-154-000-038-001, R-154-000-191-112, R-154-000-404-112, R-154-000-553-112, R-154-000-565-112, R-154-000-630-112, R-154-000-A08-592, R-154-000-A27-597, R-154-000-A91-592, R-154-000-A95-592, R-154-000-B99-114, BMRC/01/1/21/18/077, BMRC/04/1/21/19/315, BMRC/APG2013/108, SIgN-06-006, SIgN-08-020, NMRC/1150/2008, OFIRG20nov-0033, NRF-MP-2020-0004, SFS_RND_SUFP_001_04, W22W3D0006, H17/01/a0/008 and APG2013/108. F.T.C. has received consulting fees from Sime Darby Technology Centre, First Resources Ltd, Genting Plantation, Olam International and Syngenta Crop Protection, outside the submitted work. Y.Y.S. has received research support from the NUS Resilience & Growth Postdoctoral Fellowships with grant number R-141-000-036-281. Y.Y.E.L. has received funding from the National University of Singapore Department of Biological Sciences President's Graduate Fellowship. All funding agencies had no role in the study design, data collection and analysis, decision to publish, or preparation of the manuscript.

## Availability of data and materials

All data used and included in this study are available from the corresponding author (F.T.C.).

## Author contributions

F.T.C. conceived and supervised the overall research study. W.Y.T. planned and conducted the experiments. W.Y.T. and Y.Y.E.L. analyzed the data and wrote the manuscript. Y.Y.S. assisted in collecting and consolidating the data. Y.H.S., and K.R. assisted in recruiting participants. All authors reviewed, revised, and approved the final manuscript.

## Ethics approval and consent

Recruitment of participants in Singapore was approved by the Institutional Review Board of the National Healthcare Group Domain Specific Review Board (B/04/055) and the Institutional Review Board of National University of Singapore (Ref-Code: 07–023, 09–256, 10–445, 13–075, B-10–343 and H-18–036). The Scientific and Ethical Review Committee (SERC) of UTAR granted the ethical approval (Ref-code: U/SERC/03/2016) for the study conducted in Malaysia. The recruitment of participants was performed in accordance with the Helsinki Declaration. Consent forms and participant information sheets were used to collect informed consent from study participants. For participants aged below 21 years old, additional consent is obtained from a parent or guardian.

## Authors’ consent for publication

All authors have read and consented to the publication of this manuscript.

## Acknowledgements

We extend our sincerest gratitude to all participants for their contributions to this study.

## Declaration of competing interest

The authors declare no competing interests.
