# Supplementary material for: Atopic dermatitis-associated genetic variants regulate LOC100294145 expression implicating interleukin-27 production and type 1 interferon signaling
Source: World Allergy Organ J. 2024 Jan 12;17(2):100869. doi: 10.1016/j.waojou.2023.100869 (PMC10827559; doi:10.1016/j.waojou.2023.100869)
Supplement: Multimedia component 1 [file mmc1.docx]

**Supplementary Methods**

**Genomic DNA extraction and Genotyping**

Buccal cell samples were collected from participants using 10 ml of 0.9% saline mouthwash. Genomic DNA was extracted from the buccal cell samples using AxyPrep™ Multisource Genomic DNA Miniprep Kit (Axygen). The extracted genomic DNA are quantified in triplicates using Nanodrop ND-1000 (Thermo Scientific). Four GWAS arrays were used to genotype 5055 samples: Infinium Global Screening Array, Illumina HumanHap 550 k BeadChip version 3, Infinium OmniZhongHua-8 v1.3 BeadChip platform and InfiniumOmni2–5Exome. The IMPUTE2 program was used for haplotype phasing and imputation. SNP-disease association is performed on compiled results from all arrays. To decrease heterogeneity in the population, only Singapore Chinese are included in the analyzed study population. The genome-wide association significance and suggestive threshold are defined at the standard p-value of 5 × 10^-8^ and 1 × 10^-5^ respectively.

**Positive Skin Prick Test Reactions**

The atopic status is defined as a positive skin prick test reaction towards the common house dust mites *Blomia tropicalis* or *Dermatophagoides pteronyssinus*. The formation of a wheal 15 minutes after the skin prick, that has a diameter of at least 3mm, is considered a positive response. Histamine was used as a positive control while saline was used as a negative control.

**Whole-transcriptome sequencing of total RNA in PBMCs**

Participants were recruited from the University of Tunku Abdul Rahman (UTAR), Malaysia and the National University of Singapore (NUS). The recruitment in UTAR took place in January and October 2018, while the recruitment in NUS occurred in August 2013. Ten milliliters of whole blood samples were collected from each individual. PBMCs were isolated from whole blood with Ficoll-Hypaque density-gradient centrifugation and the E.Z.N.A.® Total RNA Kit from Omega Bio-tek Inc. was used to extract total RNA for whole-transcriptome sequencing. Illumina NovaSeq 6000 platform was used to perform next-generation sequencing. Mapping of raw sequences to the human genome assembly (NCBI GRCh38) and the calculation of Fragments per Kilobase of transcript per Million mapped reads (FPKM) were carried out using TopHat version 2.1.1 and Cufflinks version 2.2.1 respectively. To eliminate batch variation, all samples were sequenced at the same time.

**Selection of Hub Genes using Cytohubba**

Top ten genes were selected in Cytohubba using three measures: Maximum Clique Centrality (MCC), Maximum Neighborhood Component (MNC) and Density of Maximum Neighborhood Component (DMNC) algorithms [1,2]. The overlapping genes in at least two of these three methods were defined as hub genes.

**Supplementary Results**

**Hub genes are upregulated in inflamed individuals**

In the stratum corneum of individuals with AD, an increased ratio of IL-1RA to IL-1A was detected [3]. Increased levels of IL-1RA was also found in the circulation during an inflammatory response [4,5]. As such, we stratified individuals based on *IL-1RA*/*IL-1A* transcript ratio into high and low inflammation samples using the median splitting method. We found that the hub genes are significantly upregulated in PBMCs of inflamed individuals, further supporting the role of type 1 IFN in AD as an inflammatory skin disease.

**References**

1. Chin CH, Chen SH, Wu HH, Ho CW, Ko MT, Lin CY. cytoHubba: Identifying hub objects and sub-networks from complex interactome. BMC Syst Biol [Internet]. 2014 Dec 8 [cited 2023 Dec 6];8(4):1–7. Available from: https://bmcsystbiol.biomedcentral.com/articles/10.1186/1752-0509-8-S4-S11

2. Shen Z, Chen Q, Ying H, Ma Z, Bi X, Li X, et al. Identification of differentially expressed genes in the endothelial precursor cells of patients with type 2 diabetes mellitus by bioinformatics analysis. Exp Ther Med [Internet]. 2019 Nov 22 [cited 2023 Dec 6];19(1):499. Available from: /pmc/articles/PMC6923743/

3. Terui T, Hirao T, Sato Y, Uesugi T, Honda M, Iguchi M, et al. An increased ratio of interleukin-1 receptor antagonist to interleukin- 1α in inflammatory skin diseases. Exp Dermatol [Internet]. 1998 Dec 1 [cited 2023 Nov 30];7(6):327–34. Available from: https://onlinelibrary.wiley.com/doi/full/10.1111/j.1600-0625.1998.tb00332.x

4. Cartmell T, Luheshi GN, Hopkins SJ, Rothwell NJ, Poole S. Role of endogenous interleukin-1 receptor antagonist in regulating fever induced by localised inflammation in the rat. J Physiol [Internet]. 2001 Feb 2 [cited 2023 Dec 12];531(1):171–80. Available from: /pmc/articles/PMC2278459/

5. Gabay C, Smith MF, Eidlen D, Arend WP. Interleukin 1 receptor antagonist (IL-1Ra) is an acute-phase protein. J Clin Invest [Internet]. 1997 Jun 15 [cited 2023 Dec 12];99(12):2930–40. Available from: https://pubmed.ncbi.nlm.nih.gov/9185517/
